# Real-world effectiveness of difelikefalin for CKD-aP in UK haemodialysis: a single-centre experience

**DOI:** 10.1093/ckj/sfag129

**Published:** 2026-04-29

**Authors:** Tiffany Tuhirman, Sabrin Ali, Shraddha Ranjan, Farid Ghalli, Victoria Ingham

**Affiliations:** Sussex Kidney Unit, Royal Sussex County Hospital, University Hospital Sussex NHS Foundation Trust, Brighton, UK; Sussex Kidney Unit, Royal Sussex County Hospital, University Hospital Sussex NHS Foundation Trust, Brighton, UK; Sussex Kidney Unit, Royal Sussex County Hospital, University Hospital Sussex NHS Foundation Trust, Brighton, UK; Sussex Kidney Unit, Royal Sussex County Hospital, University Hospital Sussex NHS Foundation Trust, Brighton, UK; Brighton and Sussex Medical School, Brighton, UK; Sussex Kidney Unit, Royal Sussex County Hospital, University Hospital Sussex NHS Foundation Trust, Brighton, UK

To the Editor,

Chronic kidney disease–associated pruritus (CKD-aP) affects approximately 40% of haemodialysis (HD) patients and is associated with increased mortality, sleep disturbance, low mood and reduced quality of life [1]. Conventional therapies—topical agents, antihistamines, gabapentinoids, phosphate binders—provide limited relief for many patients [1]. Difelikefalin (DFK), a selective kappa-opioid receptor agonist, received National Institute for Health and Care Excellence (NICE) TA890 approval in 2023 for moderate–severe CKD-aP where usual antipruritic therapies have proven inadequate [2]. KALM-1/2 phase 3 trials reported 51% ≥3-point Worst Itch Numeric Rating Scale (WI-NRS)among responders taking DFK versus 28% who received placebo, and somnolence in 5%–7% of patients, with discontinuation rates of ∼8% [3, 4]. Despite demonstrated efficacy, UK adoption of DFK remains variable, likely reflecting limited real-world evidence, BlueTeq approval processes involving national prior-approval forms for high-cost medicines and inconsistent itch assessment practices.

This Caldicott Guardian–approved service evaluation, providing local data protection oversight in UK healthcare, was conducted in accordance with UK Health Research Authority guidance and did not require formal ethics approval. It had three aims: to assess the real-world effectiveness and tolerability of DFK; to quantify itch burden and treatment gaps in routine HD practice; and to develop practical implementation tools for network-wide use.

A retrospective review of CV5 (Sussex Kidney Centre electronic clinical records) prescribing logs identified 18 DFK initiations (October 2023–November 2025) among 762 in-centre HD patients; 17 were evaluable following one death exclusion. Telephone interviews in December 2025 (100% completion, *n* = 17) captured recalled pre-DFK WI-NRS (0–10 worst 24-h itch), current WI-NRS (≥4 weeks after DFK 0.5 µg/kg intravenously post-HD three times weekly; 50% >12 weeks), categorical response and adverse effects (drowsiness, diarrhoea, dizziness and falls). Demographic and clinical data were extracted from electronic records. Additionally, an anonymous patient survey (60/134 responses, 45% rate) used validated WI-NRS, 5-D Itch Scale and Dermatology Life Quality Index (DLQI) to quantify itch burden. Descriptive statistics were performed using Excel, with paired comparisons analysed using the Wilcoxon signed-rank test.

Among 14 patients continuing DFK, median WI-NRS decreased from 10.0 [interquartile range (IQR) 9.3 to 10.0] to 5.0 (IQR 3.0 to 7.3) with a median change of –5.5 (IQR –7 to –1.5) (*P* = .0015) (Fig. 1). The mean WI-NRS reduction was 4.4 points (95% confidence interval –6.8 to –2.0). Clinically meaningful response (≥3-point reduction, per KALM trials) was achieved by 71.4% (10/14); 78.6% reported improvement, and 7.1% achieved complete remission (WI-NRS = 0). DFK was generally well tolerated, with 88% (15/17) reporting no adverse events. The three discontinuations (3/17) were due to kidney transplant (*n* = 1), drowsiness despite good response (*n* = 1) and undocumented reason (*n* = 1).

**Figure 1:**
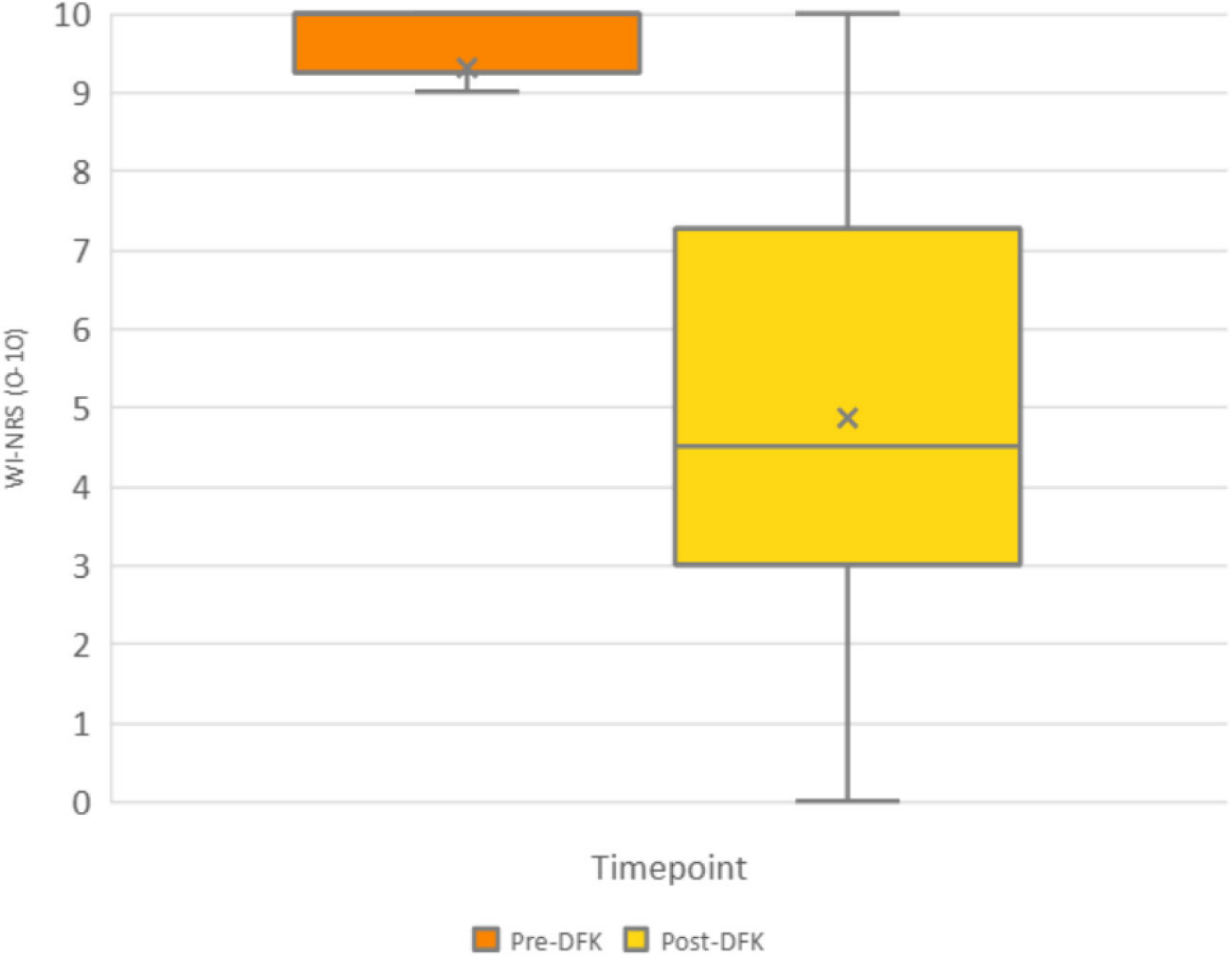
WI-NRS before and after DFK among 14 continuing patients. Median WI-NRS reduced from 10.0 (IQR 9.3 to 10.0) to 5.0 (IQR 3.0 to 7.3), with overall median change –5.5 (IQR –7 to –1.5) (*P* = 0.0015, Wilcoxon signed-rank test). Mean WI-NRS decreased from 9.3 ± 1.4 to 4.9 ± 3.1 (mean change –4.4 ± 4.1, 95% confidence interval –6.8 to –2.0).

The anonymous itch burden survey (60/134 responses, 45% response rate; mean age 67 years) revealed substantial unmet need: mean WI-NRS 3.45 (median 2.5; 47% ≥4, moderate–severe and DFK-eligible); 5-D Itch Scale 10.8 (moderate impact); and DLQI 5.9 (small–moderate quality-of-life impairment). Approximately 40% reported co-existing skin conditions (predominantly xerosis and eczema) and 45% used antipruritics, yet 76% experienced only little or modest benefit. The most common therapies were phosphate binders (43%), antihistamines (32%), and emollients or gabapentinoids (13% each). Nearly half (45%) expressed interest in DFK.

We developed a four-component implementation toolkit, now deployed across the network: (i) CKD-aP Management Flowchart to standardize WI-NRS based assessment and eligibility for DFK; (ii) DFK Standard Operating Procedure (SOP) (pending governance approval); (iii) CV5 Electronic Documentation Template; and (iv) Patient Information Leaflet.

This UK real-world evaluation demonstrates that DFK has substantial effectiveness and good tolerability in routine HD practice, with response rates comparable to pivotal trial benchmarks. Despite 47% moderate–severe CKD-aP prevalence and 76% conventional therapy failure, implementation barriers like BlueTeq approval processes persist. Structured implementation tools may facilitate systematic WI-NRS screening, standardized prescribing and audit-ready monitoring, supporting practical operationalization of NICE TA890 and improving equitable access across UK renal networks.

## References

[1] Agarwal R, Rayner HC, Al-Ghonaim M et al. Alleviating symptoms in patients undergoing long-term haemodialysis: a focus on chronic kidney disease-associated pruritus. Clin Kidney J. 2023;16:30–40. 10.1093/ckj/sfac187 36726430 PMC9871858

[2] National Institute for Health and Care Excellence . Difelikefalin for treating pruritus in people having haemodialysis (TA890). London, UK: NICE, 2023. 40138504

[3] Fishbane S, Jamal A, Munera C et al. A phase 3 trial of difelikefalin in haemodialysis patients with pruritus. N Engl J Med. 2020;382:222–32. 10.1056/NEJMoa1912770 31702883

[4] Fishbane S, Mathur VS, Kothare PA et al. Difelikefalin versus placebo for chronic pruritus in patients undergoing haemodialysis (KALM-2). Lancet. 2020;396:1449–60.

